# Prevalence of Posttraumatic Stress Disorder in Persons with Chronic Pain: A Meta-analysis

**DOI:** 10.3389/fpsyt.2017.00164

**Published:** 2017-09-14

**Authors:** Johan Siqveland, Ajmal Hussain, Jonas Christoffer Lindstrøm, Torleif Ruud, Edvard Hauff

**Affiliations:** ^1^Department for Research and Development, Division of Mental Health Services, Akershus University Hospital, Lørenskog, Norway; ^2^Regional Resource Centre for Traumatic Stress and Suicide Prevention, Oslo, Norway; ^3^Institute of Clinical Medicine, University of Oslo, Oslo, Norway; ^4^Health Services Research Unit, Akershus University Hospital, Lørenskog, Norway; ^5^Oslo University Hospital, Oslo, Norway

**Keywords:** posttraumatic stress disorder, PTSD, chronic pain, systematic review, meta-analysis, prevalence

## Abstract

**Objective:**

To summarize evidence for the prevalence of posttraumatic stress disorder (PTSD) among persons with chronic pain (CP).

**Methods:**

We searched databases for studies published between January 1995 and December 2016, reporting the prevalence of PTSD in persons with CP. Two reviewers independently extracted data and assessed the risk of bias. We calculated the pooled prevalence using a random-effects model and performed subgroup analyses according to pain location, the population and assessment method.

**Results:**

Twenty-one studies were included and the PTSD prevalence varied from 0–57%, with a pooled mean prevalence of 9.7%, 95% CI (5.2–17.1). In subgroup analysis, the PTSD prevalence was 20.5%, 95% CI (9.5–39.0) among persons with chronic widespread pain, 11.2%, 95% CI (5.7–22.8) among persons with headache, and 0.3%, 95% CI (0.0–2.4) among persons with back pain. The prevalence in clinical populations was 11.7%, 95% CI (6.0–21.5) and in non-clinical populations 5.1%, 95% CI (0.01–17.2). In studies of self-reported PTSD symptoms, PTSD prevalence was 20.4%, 95% CI (10.6–35.5), and in studies where structured clinical interviews had been used to assess PTSD its prevalence was 4.5%, 95% (CI 2.1–9.3). The risk of bias was medium for most studies and the heterogeneity was high (*I*^2^ = 98.6).

**Conclusion:**

PTSD is overall more prevalent in clinical cohorts of persons with CP and particularly in those with widespread pain, but may not always be more prevalent in non-clinical samples of persons with CP, compared to the general population. There is a large heterogeneity in prevalence across studies. Future research should identify sources of heterogeneity and the mechanisms underlying the comorbidity of the two conditions.

## Background

Chronic pain (CP) is among the most common and costly health problems in the West (1, 2). A recent epidemiological study in 16 countries (3) found that 19% of the general population has CP, two-thirds of these describe their pain as moderate and one-third as severe and more women than men report having CP, according to this study. Mental illnesses, such as anxiety disorders and depression, are more prevalent among persons with CP (3–5), and there is evidence that anxiety disorders could be more strongly associated with painful conditions than depression (6). Mental disorders are associated with more functional impairment in persons with CP (5) and may, therefore, be a negative prognostic factor for recovery (7). So, an improved understanding of the prevalence of mental disorders associated with CP is important for the planning and dissemination of effective CP treatment (8). Posttraumatic stress disorder (PTSD) is a relatively common anxiety disorder developing in some people after exposure to traumatizing, unusual events, such as accidents, violence, or war. PTSD is characterized by the three symptom clusters: (i) hyperarousal, (ii) intrusions of memories from the traumatic event, and (iii) avoidance of situations and actions reminiscent of the event (9). The 12-month PTSD prevalence in the general population is reported to be 3.6% in the United States (10) and 1.1% in Europe (11). As for CP, there are gender differences in the risk of developing PTSD after exposure to trauma and in the PTSD prevalence. Women have an increased risk of developing PTSD after exposure to both man-made and technological disasters (12), and more women than men have PTSD with an odds ratio (OR) of 2.69 (13). The results of numerous clinical epidemiological studies have led to the conclusion that PTSD is more common in persons experiencing different forms of CP, such as fibromyalgia (FM) (14) and migraine (15). Previous research indicates that PTSD is related to CP in both clinical samples and samples of the general population. High rates of PTSD have been reported in clinical subjects seeking treatment for CP (16), and in one study, which included a large sample, it was found that CP was related to PTSD with an OR of 3.69 (5). Added to this, PTSD comorbidity with CP is a complicating factor for its treatment and results in the efficacy of therapeutic interventions being lower (17).

The reasons for the often-reported association between PTSD and CP are not very well understood, but some theoretical models have been proposed. One model suggest that the relationship is related to a shared vulnerability for developing both PTSD and CP (18, 19) while others have suggested that the conditions mutually maintain each other (20) and a recent review summarized possible neurobiological and neuroanatomical models that might explain this association (21).

While the causes for the association between PTSD and other anxiety disorders are still to a large extent unknown, there has been a growing research interest in describing the relationship between mental disorders and somatic syndromes, including CP, and some recent systematic reviews have summarized the available published research. The findings from three previous systematic reviews and meta-analyses represent relevant background material for the present review. A review of the relationship between psychological trauma and functional somatic syndromes (FSS) looked at three CP disorders: FM, chronic widespread pain (CWP), and temporomandibular disorder (22). The review included a meta-analysis of the results from 71 studies comparing FSS in persons exposed to psychological traumas to non-exposed control individuals and found that exposure to trauma was related to a 2.7 times increase in the risk of developing FSS. The review authors noted that the included studies were of low overall quality and this may have affected the findings and that authors using validated measures of trauma exposure reported a weaker association between trauma exposure and FSS. The association between trauma exposure and FSS, therefore, could be below the pooled estimate from this meta-analysis.

A later study corroborated that there is a positive relationship between exposure to trauma and pain in a meta-analysis of 18 case–control studies into the association between exposure to emotional, physical and sexual abuse, and FM. In this review, a diagnosis of FM was found to be significantly associated with childhood abuse with an OR of 2.49. Finally, in a meta-analysis study, Fishbain et al. (23) analyzed the relationship between PTSD and CP. This review includes 40 studies, and the prevalence of PTSD in them ranged between 0.69% in a group of persons with lower back pain and 50.1% in a group of war veterans.

The aims of the present study were to assess the prevalence of PTSD in persons with CP and to determine how it varies according to the location of the pain, the population, and the methods used to assess the diagnosis.

## Methods

We followed the quality standards for systematic reviews as presented in the PRISMA statement, which is attached as Supplementary material. The review protocol is also attached as Supplementary material.

### Search Strategy

We searched for articles published in the English language after January 1995 and listed in the PsycINFO, MEDLINE, and PubMed databases. January 1995 is when the first studies based on the DSM IV diagnosis handbook could have emerged. The search was last updated in November 2016. We used a number of textword combinations related to CP, such as “chronic pain,” “migraine,” “chronic daily headache,” “fibromyalgia,” “widespread pain,” “musculoskeletal pain,” “rheumatoid pain,” “chronic back pain,” and “chronic spinal pain” combined with textword for posttraumatic stress “Stress Disorders,” “post-traumatic/,” and “PTSD.” In addition, all the reference lists of the studies included were hand searched for further relevant publications. The search string for the search in the Embase, MEDLINE, and PsycINFO databases is attached as Supplementary material.

### Inclusion of Studies

After eliminating all duplicate studies, two reviewers independently examined the titles and abstracts of all the extracted articles. We retrieved the full texts of articles that were considered, by at least one reviewer, to meet the inclusion criteria. The inclusion criteria were studies published in English reporting the point prevalence (within 1 month) of PTSD based on a structured assessment according to the PTSD criteria of the DSM IV or the International Statistical Classification of Diseases and Related Health Problems (10th Edition; ICD-10). Exclusion criteria were military-only samples, samples recruited from a mental health-care setting, studies using non-structured assessments (such as informal clinical interviews) or assessments that did not represent sufficiently the diagnostic criteria for PTSD. Migraine was considered a CP disorder, based on the assumption that it is a chronic disorder, despite being characterized by episodic presentation of symptoms (24). After retrieving the full texts, the reviewers extensively scrutinized them to confirm the eligibility of the studies based on the above criteria. Any disagreements relating to inclusion eligibility were resolved through discussions.

### Data Extraction

The reviewers (Ajmal Hussain and Johan Siqveland) extracted information about sample size, number of participants with PTSD, pain location, the setting of recruitment, the method of PTSD assessment, and nationality. Both reviewers agreed on the final data extraction, ensuring accuracy, and disagreements concerning data selection were resolved *via* discussion between them.

### Risk of Bias Assessment

We assessed the risk of bias using an 8-item scale (range 0–8; see Supplementary Material) adapted for this study from the EBMH Notebook list for evaluating risk of bias in prevalence studies (25). This scale comprises various criteria for evaluating sources of bias, such as the quality of the diagnostic assessments and sample size. We gave the studies included one point for each criterion met, with higher scores, therefore, indicating a lower risk of bias. Studies that scored a seven or eight were classified as having a low risk of bias. Those scoring between three and six, and those scoring between 0 and 2, were of medium and high risk, respectively.

### Statistical Analysis

We hypothesized that the prevalence of PTSD would vary according to pain location, assessment method, and population, so we performed three analyses of subgroups. In the first subgroup analysis, studies included were grouped according to pain location: (a) headaches, including migraines, tension-type headaches, chronic daily headaches, and other headaches; (b) back pain, including pain originating from the spine and chronic lower back pain; (c) CWP, according to the American College of Rheumatology criteria of widespread pain or FM syndrome; and (d) other CP conditions including peripheral neuropathic pain or mixed CP. In the second subgroup analysis, we divided studies according to the population studied: (a) participants recruited from clinical settings and (b) participants from non-clinical settings. Finally, in the third subgroup analysis, we grouped the studies according to the assessment method used: (a) self-reporting of assessed PTSD symptoms and (b) a structured clinical interview for assessing a PTSD diagnosis.

As we had expected there to be significant heterogeneity across the studies included, we used a random-effects model. We analyzed the prevalence of PTSD using a binomial-normal random-effects meta-regression model (26) in the “metafor” R package (27). A random-effects model allows for explicit modeling of the heterogeneity of the parameters investigated in each study, assuming that the true logit transformed prevalence rates are normally distributed among the studies. We calculated the 95% confidence interval (95% CI) for all estimates. Heterogeneity was assessed and reported using the *I*^2^ statistic, which describes the variance between studies as a proportion of the total variance. The *I*^2^ statistic varies between 0 and 100%, with 0% being interpreted as no heterogeneity, 25% as low heterogeneity, 50% as medium heterogeneity, and 75% and above as a high degree of heterogeneity (28).

We categorized the studies according to pain location, population, and assessment, including them in the model as categorically coded predictors in three separate sub-analyses. Finally, we estimated the regression coefficients and transformed them into prevalence rates for easier interpretation and reporting.

## Results

### Overview of Search

**Figure 1:**
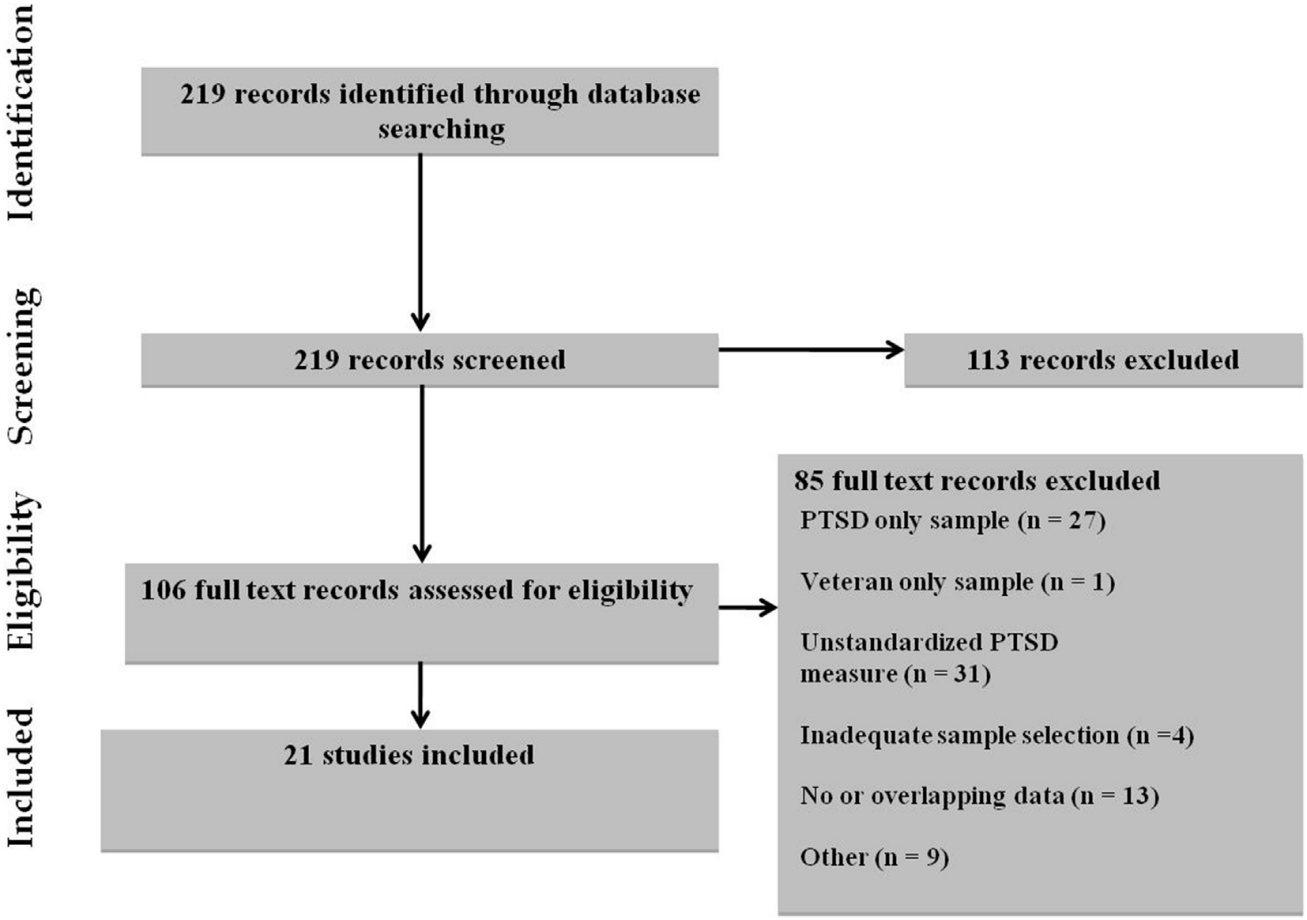
Selection of studies.

After excluding duplicates, the literature search yielded 1,589 records. In the initial screening of abstracts, 1,473 articles were excluded, as they did not meet the inclusion criteria. After reading the full texts, we excluded 95 studies the reasons for this presented in Figure 1.

**Figure 2:**
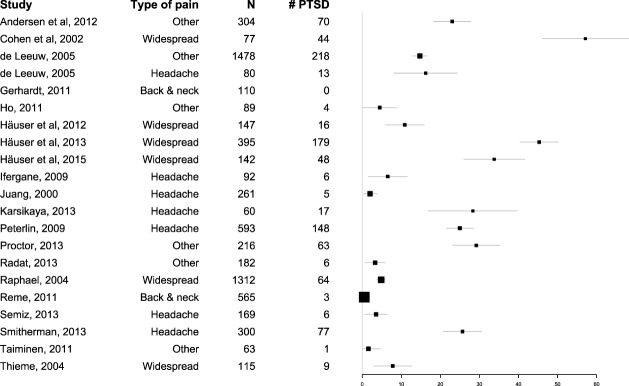
Forest plot of the Prevalence of PTSD Among Persons with Chronic Pain.

The prevalence of PTSD in the studies included is given in Table 1. A forest plot depicting the prevalence of PTSD in the included studies is presented in Figure 2.

**Table 1:** Selected characteristics, PTSD prevalence, and risk of bias of the included studies.

Reference	Country	Sample selection	Type of pain	Assessment instrument	*n* (% women)	PTSD prevalence (%)	Trauma exposure	Risk of bias score
Andersen et al. (16)	Finland and Denmark	Multidisciplinary pain center	Mixed chronic pain (CP)	HTQ	304 (60.5)	23.0	84% reported > 1 sign. stressor	5
Cohen et al. (29)	Israel	Rheumatology clinic	Fibromyalgia (FM)	SCID	77 (51.9)	57.0	Mean exposure 5.7 events	4
de Leeuw et al. (30)	United States	Orofacial pain center	Orofacial pain	PCL-C	1,478 (86.4)	15.0	NA	4
de Leeuw et al. (31)	United States	Orofacial pain center	TTH or migraine	PCL-C	80 (78.8)	16.3	64% reported > 1 sign. stressor	3
Gerhardt et al. (32)	Germany	General population	Low back pain	SCID	110 (57.3)	0.0	NA	6
Ho et al. (33)	China	CP clinic	Mixed CP	SCID	89 (56,2)	4.5	NA	6
Häuser et al. (34)	Germany	General population	Widespread pain	PDS	147 (52)	10.9	49% reported > 1 sign. stressor	5
Häuser et al. (14)	Germany	General population	FM	PDS	395 (93.9)	45.3	74.5% reported > 1 sign. stressor	7
*Häuser* et al. (35)	Germany and United States	Rheumatology clinic/pain medicine center	FM	PDS	142 (95.8)	33.8	60.3% reported > 1 sign. stressor	6
Ifergane et al. (36)	Israel	Headache clinic	Migraine	CAPS	92 (77.2)	6.5	16.3% reported > 1 sign. stressor	6
Juang et al. (37)	Taiwan	Headache clinic	CDH	MINI	261 (80)	2.0	NA	6
Karsikaya et al. (38)	Turkey	Neurology clinic	Migraine	SCID	60 (73,3)	28.3	63.3% reported > 1 sign. stressor	7
Peterlin et al. (39)	United States	Headache center	Migraine	PCL-C	593 (92)	25.0	81.2% reported > 1 sign. stressor	6
Proctor et al. (40)	United States	Neurodiagnostic clinic	Pain related to industrial injuries	CAAPE	216 (51.9)	29.2	NA	5
Radat et al. (41)	France	Neurological and pain centers	Neuropathic pain	MINI	182 (52.2)	3.3	NA	7
Raphael et al. (42)	United States	General population	FM	PCL-C	1,312 (100)	4.8	All participants in vicinity of New York at 9/11	4
Reme et al. (43)	Norway	Primary care patients	Low back pain	MINI plus	565	0.6	NA	6
Semiz et al. (44)	Turkey	Students	Migraine	SCID	169 (45.7)	3.5	NA	6
Smitherman et al. (15)	United States	Students	Migraine	PCL-C	300 (63.1)	25.7	69.3% reported one or more significant stressors	4
Taiminen et al. (45)	Finland	Neurology or oral diseases clinic	Mixed CP	SCID	63 (90)	1.6	NA	5
Thieme et al. (46)	Germany	Rheumatology department	FM	SCID	115 (100)	7.8	40.9% experienced physical abuse 20.9% experienced sexual abuse	5

*NA, not available; CDH, chronic daily headache; TTH, tension-type headache; HTQ, Harvard Trauma Questionnaire; SCID, structured clinical interview for DSM; PCL-C, posttraumatic checklist civilian version; PDS, Posttraumatic Diagnostic Scale; CAPS, Clinician Administered PTSD Scale; MINI, M.I.N.I. International Neuropsychiatric Interview; CAAPE, comprehensive assessment, and psychological evaluation*.

Twenty-one articles with a total of 6,750 participants fulfilled the inclusion criteria and were included in the analysis. These 21 studies varied in sample size between 60 patients with migraine (38) and 1,478 patients with orofacial pain (30). Six of the studies included participants with widespread pain, seven studies included participants with headaches, and two studies included participants with back pain. Six studies included participants with other pain conditions or mixed pain. Three structured or semi-structured diagnostic interviews to diagnose PTSD were used in 10 of the studies, with four questionnaires used in 11 studies. All studies made diagnostic categorization according to the DSM IV criteria for PTSD.

### Prevalence of PTSD in All Studies

The pooled prevalence across all studies was 9.7% (95% CI 5.2–17.1). The lowest prevalence was reported in a population-based study of persons with lower back pain (*n* = 110), where none of the participants had PTSD (32). In contrast, the highest prevalence was reported in a study of patients with FM (*n* = 77), where 57% of participants had PTSD (29).

### Prevalence of PTSD According to Pain Location, the Study Population, and the Assessment Method Used

The prevalence rates of PTSD with 95% CIs and heterogeneity statistics according to the location of the pain, the study population, and the assessment method are presented in Table 2.

**Table 2:** Pooled estimate and 95% CI by location of pain, assessment, and population.

	Cohorts	Participants	Pooled PTSD prevalence	95% CI	*I*^2^ (%)
**Pain location**					
Back	2	675	0.3	0.0–2.4	^a^
Head	7	1,555	11.2	5.1–22.8	96.2
Widespread	6	2,209	20.5	9.5–39.0	98.0
Other/mixed	6	2,339	9.1	3.8–20.5	97.5
**Assessment**					
Questionnaire	10	4,988	20.4	10.6–35.5	94.2
Interview	11	1,790	4.5	2.1–9.3	97.2
**Sample**					
Non-clinical	5	2,059	5.1	1.4–17.2	97.4
Clinical	16	4,719	11.7	6.0–21.5	98.6
Total	21	6,778	9.7	5.2–17.1	98.6

*^a^Not applicable due to low number of cohorts*.

The seven studies of persons suffering with headache (*n* = 1,555) indicated a PTSD prevalence ranging from 2.0 to 28.3%, with a pooled estimate of 11.2% (95% CI 5.1–22.8%). The six studies of persons with CWP (*n* = 2,189) indicated a PTSD prevalence from 4.8 to 57.0%, with a pooled estimate of 20.5% (95% CI 9.5–39.0%). The two studies on persons with back pain (*n* = 675) demonstrated PTSD prevalence rates of 0 and 0.6%, respectively, with a pooled estimate of 0.3% (95% CI 0.0–2.4%). The six studies including other pain conditions or mixed pain conditions reported PTSD prevalences from 1.6 to 29.2%, with a pooled estimate of 9.1% (95% CI 3.8–20.5%).

The 16 studies of clinical samples (*n* = 4,719), reported PTSD prevalences from 0.6 to 57%, pooled estimate 11.7% (95% CI 6.0–21.5%). The five studies with non-clinical samples (*n* = 2,059), reported PTSD prevalences from 0 to 25.7%, pooled estimate 5.1% (95% CI 1.4–17.2%).

The 11 studies where PTSD was assessed with a structured clinical interview (*n* = 1,790) indicated PTSD prevalence rates from 0.6 to 57%, pooled estimate 4.5% (95% CI 2.1–9.3%). The 10 studies that included self-reported assessments of PTSD (*n* = 4,988) reported a PTSD prevalence from 4.8 to 45.8%; pooled estimate 20.4% (95% CI 10.6–35.5%).

### Analysis of Heterogeneity

The total heterogeneity in the included studies was very high (*I*^2^ = 98.6). To understand further this large variation in the included we also calculated heterogeneity estimates in the subgroup analysis based on location of pain, population, and assessment method. However, this did not reduce the heterogeneity substantially (*I*^2^ 94.2–98.6). Because PTSD is more common in women than it is in men (47), we also analyzed the studies with gender as a co-variate. However, we found that this did not reduce heterogeneity either (*I*^2^ = 98.3).

### Risk of Bias Assessment

In Table 1, scores for the risk of bias are presented. Of the 21 studies included here, three had a low risk of bias and 18 had a medium risk. The lack of comparison between responders and non-responders, unclear exclusion criteria, and self-reported PTSD symptoms were the most common causes for the higher levels of risk of bias.

## Discussion

The pooled PTSD prevalence across the 21 studies included in the current analysis was 9.7% (95% CI 5.2–17.1%), but varied widely according to pain location, the assessment method, and the sample selection. This finding, however, is in line with a recently published systematic review of PTSD in CP using other selection criteria, which reported a prevalence of 9.8% (23). Despite the seemingly similar inclusion criteria, 14 out of the 21 studies included in this meta-analysis are unique to the present study and were not included in the study by Fishbain et al. (20). There were several reasons for this low overlap in the included studies in these two reviews. In contrast to the other review article, we only included studies published after the introduction of DSM IV for more homogenous diagnostic evaluations, we excluded studies recruiting participants from a military setting and we included only studies assessing current PTSD.

Overall, the studies included indicated a higher prevalence of PTSD in persons with CP than that in the general population [between 1 and 4% (11, 48)]. Here, analysis of the subgroups showed that a higher prevalence of PTSD might be limited to some CP locations and populations, and may possibly be confounded by the assessment methods.

The prevalence of PTSD varied significantly according to the pain location and was most prevalent in persons with widespread pain. The prevalence of PTSD was lowest in the two studies of back pain. The PTSD prevalence in these two studies was not significantly higher than that determined in studies of the general population. The low PTSD prevalence in studies of back pain indicates that back pain may be less closely associated with PTSD, compared to other types of pain. Back pain is the most common type of CP (3) and may be more related to general lifestyle factors rather than to psychological factors. However, there is an alternative, methodological explanation to the low prevalence of PTSD in the back pain studies. The two included studies on back pain both were European diagnostic interview studies with participants from the general population (32) or from primary care (43) rather than from specialist pain clinics. Both assessment type, geographical area for recruiting participants and population are factors related to a lower PTSD prevalence compared to studies based on symptom self-reporting, studies carried out in the USA (11, 48) and studies with clinical populations. These three factors might have exaggerated the real difference in the prevalence PTSD between back pain sufferers and those with other types of pain.

Pain that was widespread was the type with the highest prevalence of PTSD. Our analysis was not suited to finding the causes for the differences in the prevalence between different pain types. However, a hypothesis for the differences can be proposed; neurobiological changes related to PTSD in the CNS and endocrine system affect pain sensitivity in the whole body, such as a reduction in the levels of neuropeptide Y, and the neuroactive steroids allopregnanolone and pregnanolone (21). The same underlying pathophysiological processes relating to both CP and PTSD might be more pronounced in persons with widespread, compared to localized, pain, as pain mechanisms mainly work in a generalized way throughout the body. The relationship between widespread pain and PTSD may also be related to common risk factors. Women are at an increased risk of having both widespread pain and PTSD and so both PTSD and widespread pain are more common in women than in men, with an OR of between 2 and 3 (13, 49). This association might be due to the exposure of women to traumatic events with the most serious health consequences. These would be interpersonal, psychological, and physical violence. Lastly, processes maintaining PTSD or pain (or both conditions) at the behavioral level may be more pronounced in persons with widespread pain. One possible mechanism is avoidance, which would play a role in mutually maintaining both pain and PTSD (50).

The prevalence of self-reported PTSD was significantly higher than the prevalence of PTSD in studies using clinical interviews for diagnosis, with pooled estimates of 20.4 and 4.5%, respectively. This finding is consistent with that of a previous review on the relationship between somatic disorders and PTSD (51). Since structured or semi-structured diagnostic interviews are the gold standard for PTSD assessments, it is highly likely that this difference is attributable to studies based on self-reporting overestimating the prevalence of PTSD. Questionnaires are often constructed for screening purposes and have cutoffs for probable PTSD diagnosis biased toward sensitivity rather than specificity (52), because this is of the greatest clinical use. The issues with over-inclusiveness may have been further accentuated in these analyses due to the self-reporting questionnaires not being validated for diagnostic accuracy in a CP population. For instance, CP patients have a higher-than-average level of avoidance and hyperarousal (53), relating to their pain disorder. These symptoms, while they are related to pain, are particularly difficult to differentiate from the symptoms of PTSD, particularly in self-report assessments. Therefore, studies using the self-reporting of PTSD in CP populations may be associated with more false positive PTSD cases, and the prevalence of PTSD reported in interview studies is more comparable to the prevalence from previous interview studies in the general population (10, 11). Previous estimates of the PTSD prevalence in CP sufferers may, have considerably overstated this comorbidity.

The data presented here show a clear tendency toward the expected difference between clinical- and population-based samples. PTSD was twice as prevalent in the clinical studies as in the studies of the general population, which was close to being significant. This indicates a putative and interesting difference between clinical and general populations with CP.

### Limitations and Risk of Bias

We applied strict inclusion criteria in terms of only including diagnoses according to the latest diagnostic manuals for mental disorders, which had been available for long enough for studies to have emerged. A later version of the DSM manual for psychiatric diagnosis, DSM 5, was published during the course of this work; however, none of the studies included here involved making diagnostic evaluations according to this newer manual. Despite the attempts to make the inclusion strict to limit heterogeneity between the studies, the meta-analysis showed that the PTSD prevalence varied widely in that there very large amounts of unexplained variance across the studies. Therefore, the pooled mean prevalence has to be interpreted cautiously. The groups included in this review were also heterogeneous in terms of type of pain conditions and degree of clear somatic origin due to the inclusion criteria in the primary studies. The difficulties with finding homogenous associations between PTSD and pain is further hindered by our lack of understanding of the causal relationship between the conditions and more research and model development on this area is clearly needed.

Few of the studies included here gave an adequate description of the recruitment process or rate of non-participation. However, this represents the most common risk of bias in these studies, and so the participants included may not be fully representative of the CP population. In addition, the number of studies, and hence the participants, included was limited. Even for the most extensively studied pain condition, headaches, only seven studies including 1,555 participants were included. Only articles written in English were included, but as the prevalence of PTSD varies between different countries, adding publications in other languages might have altered our findings. Finally, as some studies included here relied on self-reported CP, not all participants in these studies may have met the formal criteria for a CP disorder.

### Clinical Implications

The identification of mental disorders in persons seeking treatment for CP may be important for several reasons. Pain treatment is often long term and requires good adherence from the patient. A diagnosis of PTSD is related to poor adherence and, therefore, it is important to assess whether this exists alongside the CP (54). The current review indicates that PTSD is prevalent in persons with CP, and particularly among persons seeking treatment for pain, and in those with widespread pain. Clinicians treating CP should be aware of the increased risk of comorbidity with PTSD in their patients, and particularly in patients with widespread pain. In addition to the risk of non-adherence, PTSD is related to an increased risk of substance use disorder (55), so the possibility for dependence needs to be weighed against the benefit for the patient before starting treatment with opioids. Interventions with proven effectiveness in the treatment of *both* PTSD and CP [such as cognitive behavioral therapy (56, 57), somatic experiencing (58), or antidepressants (59, 60)] may be considered as the first treatment in persons with comorbid pain and PTSD.

## Availability of Supporting Data

All data and material are available upon request to the first author.

## Ethics Statement

As this was meta-analysis of published data no ethics committee approval was needed.

## Author Contributions

JS and EH planned and designed the study, AH and JS screened studies for inclusion and extracted data. JL planned and performed the statistical analysis, and TR participated in drafting the article and revised it critically. All authors have given final approval of the version of the manuscript submitted.

## Conflict of Interest Statement

The authors declare that the research was conducted in the absence of any commercial or financial relationships that could be construed as a potential conflict of interest.

## Abbreviations

CP, chronic pain; PTSD, posttraumatic stress disorder; ICD-10, international classification of diseases 10th revision; DSM IV, diagnostic and statistical manual of mental disorders 4th edition; CI, confidence interval.
