# Supplementary material for: Prevalence of Posttraumatic Stress Disorder in Persons with Chronic Pain: A Meta-analysis
Source: Front Psychiatry. 2017 Sep 14;8:164. doi: 10.3389/fpsyt.2017.00164 (PMC5603802; doi:10.3389/fpsyt.2017.00164)
Supplement: Supplementary file 1 [file data_sheet_1.docx]

# Prevalence of Post-traumatic stress disorder (PTSD) in persons with chronic pain: A Meta-analysis

**Review question:** To review and assess the prevalence of posttraumatic stress disorder in persons with of chronic pain. To further perform sub group analysis according to type of chronic pain, selection of participants and how PTSD diagnosis were made.

## Searches:

We will search the PsycINFO, MEDLINE and PubMed databases.for articles published in English after January 1995 using combinations of keywords relevant for chronic pain and PTSD such as “chronic pain”, “migraine”, “chronic daily headache”, “fibromyalgia”, “widespread pain”, “musculoskeletal pain”, “rheumatoid pain”, “chronic back pain” and “chronic spinal pain. We will hand search the identified studies for further relevant studies.

**Study inclusion:**

After eliminating all duplicate studies, two reviewers independently will examine the titles and abstracts of all the extracted articles and read the full texts of articles that are considered, to meet the inclusion criteria by one of the reviewers. We will include all studies published in English reporting the point prevalence (within one month) of PTSD based on a structured assessment according to the PTSD criteria of the DSM-IV or the International Statistical Classification of Diseases and Related Health Problems (Tenth Edition; ICD-10).

Exclusion criteria:

- Military-only samples,
- Samples recruited from a mental health care setting
- Studies using non-structured assessments or assessments that did not represent sufficiently the diagnostic criteria for PTSD.

After retrieving the full texts, the reviewers will finally decide the eligibility of the studies based on the above criteria. Any disagreements relating to inclusion eligibility will be resolved through discussions.

## Data extraction

Two reviewers will independently extract information about sample size, number of participants with PTSD, pain location, the setting of recruitment, the method of PTSD assessment and nationality of the study. Disagreements concerning data selection will be resolved via discussion between them.

## Risk of bias assessment

We will assess the risk of bias using an 8-item scale being adapted for this study from the EBMH Notebook list.

**Data synthesis**

All the data will be analysed in a meta- analysis and described narratively.
