# Supplementary material for: Prevalence of Posttraumatic Stress Disorder in Persons with Chronic Pain: A Meta-analysis
Source: Front Psychiatry. 2017 Sep 14;8:164. doi: 10.3389/fpsyt.2017.00164 (PMC5603802; doi:10.3389/fpsyt.2017.00164)
Supplement: Supplementary file 3 [file data_sheet_3.doc]

**Supplementary material**

**Search strategy**

Date: November 3. 2016

Databases: Embase, Ovid MEDLINE(R), PsycINFO

Search Strategy:

1. posttraumatic stress disorder.tw
2. PTSD.tw
3. Chronic pain.tw.
4. migraine.tw.
5. chronic daily headache.tw.
6. fibromyalgia.tw.
7. widespread pain.tw.
8. musculoskeletal pain.tw.
9. rheumatoid pain.tw.
10. chronic back pain.tw.
11. chronic spinal pain.tw.
12. 1 or 2 and 3 or 4 or 5 or 6 or 7 or 8 or 9 or 10 or 11
13. Limit 12 to yr = «1994 –Current»
14. remove dublicates from 13
