# Supplementary material for: Prevalence of Posttraumatic Stress Disorder in Persons with Chronic Pain: A Meta-analysis
Source: Front Psychiatry. 2017 Sep 14;8:164. doi: 10.3389/fpsyt.2017.00164 (PMC5603802; doi:10.3389/fpsyt.2017.00164)
Supplement: Supplementary file 4 [file data_sheet_4.docx]

# Risk of bias assessment scale

| Risk of bias | **Yes = 1** | **No = 0** |
| --- | --- | --- |
| 1. **Sociodemographic characteristics are described (e.g., age/gender)** |  |  |
| 1. **Was the sample a consecutive sample?** |  |  |
| 1. **Inclusion and exclusion criteria are clear** |  |  |
| 1. **Participation or response rates described** |  |  |
| 1. **Comparison of characteristics of responders and non-responders** |  |  |
| 1. **Chronic pain is assessed by clinician using accepted methods** |  |  |
| 1. **Clinician-assessed PTSD** |  |  |
| 1. **Sample size >100** |  |  |
